# MicroRNA-126 affects rheumatoid arthritis synovial fibroblast proliferation and apoptosis by targeting *PIK3R2* and regulating PI3K-AKT signal pathway

**DOI:** 10.18632/oncotarget.12487

**Published:** 2016-10-06

**Authors:** Yuan Qu, Jing Wu, Jia-Xin Deng, Yu-Ping Zhang, Wan-Yi Liang, Zhen-Lan Jiang, Qing-Hong Yu, Juan Li

**Affiliations:** ^1^ Department of Internal Medicine of Traditional Chinese Medicine, College of Traditional Chinese Medicine, Southern Medical University, Guangzhou 510510, Guangdong, P. R. China; ^2^ Department of Rheumatology and Clinical Immunology, Zhujiang Hospital of Southern Medical University, Guangzhou 510282, Guangdong, P. R. China; ^3^ Department of Rheumatology, Nanfang Hospital, Southern Medical University, Guangzhou 510510, Guangdong, P. R. China

**Keywords:** microRNA-126, PIK3R2, PI3K-AKT, rheumatoid arthritis, synovial fibroblasts

## Abstract

Rheumatoid arthritis (RA) is a chronic autoimmune disease that causes inflammation and destruction of the joints as well as an increased risk of cardiovascular disease. RA synovial fibroblasts (RASFs) are involved in the progression of RA and release pro-inflammatory cytokines. On the other hand, microRNAs (miRs) may help control the inflammatory response of immune and non-immune cells. Therefore, our study used lentiviral expression vectors to test the effects of miR-126 overexpression on RASF proliferation and apoptosis. Luciferase experiments verified the targeting relationship between miR-126 and *PIK3R2* gene. The co-transfection of anti-miR-126 and PIK3R2 siRNA to RASFs were used to identify whether *PIK3R2* was directly involved in proliferation and apoptosis of miR-126-induced RASFs. Real-time polymerase chain reaction (PCR) was used to detect miR-126 and *PIK3R2* expressions. MTT assay was used to detect cell proliferation. Flow cytometry was used to detect cell apoptosis and cell cycle. Western blotting was used to detect PIK3R2, PI3K, AKT and p-AKT proteins. After Lv-miR-126 infected RASFs, the relative expression of miR-126 was significantly enhanced. MiR-126 promoted RASF proliferation and inhibited apoptosis. Levels of PIK3R2 decreased while total PI3K and p-AKT levels increased in RASFs overexpressing miR-126. Co-transfection of anti-miR-126 and PIK3R2 siRNA also increased PI3K and p-AKT levels as well as RASF proliferation and reduced apoptosis, as compared to anti-miR-126 treatment alone. Finally, luciferase reporter assays showed that miR-126 targeted *PIK3R2*. Our data indicate that miR-126 overexpression in RASFs inhibits *PIK3R2* expression and promotes proliferation while inhibiting apoptosis. This suggests inhibiting miR-126 may yield therapeutic benefits in the treatment of RA.

## INTRODUCTION

Rheumatoid arthritis (RA), as a chronic and autoimmune disease of the joints, affects approximately 0.5-1% of adults worldwide [1, 2]. About 30% of RA patients would become permanently work disabled in the first 2-3 years without sufficient treatment [3]. An improved understanding of RA pathogenesis has led to various modern therapeutic options; however, more research is needed to reduce side effects and improve the quality of life of patients [4]. RA synovial fibroblasts (RASFs) are major effectors of joint destruction and joint inflammation and play a critical role in the pathogenesis of RA, contributing to the formation of rheumatoid pannus [5]. RASFs participate in RA initiation, progression, and perpetuation [6]. RASFs can spontaneously secrete numerous pro-inflammatory cytokines, as well as innate immunity and matrix-degradation products, which promote RA pathogenesis [7].

Recently, microRNAs (miRs) have been proposed to be implicated in controlling the inflammatory response of immune and non-immune cells [8]. MiR-126 is located within the 7th intron of epidermal growth factor-like domain 7 and contributes to angiogenesis, proliferation, cell survival migration and invasion in some cancers [9, 10]. Additionally, miR-126 may be involved in the initiation and development of systemic lupus erythematosus by inhibiting interferon production [11]. PIK3R2 has two leading regulatory subunits, p85α and p85β, which share core functions but display unique activities [12]. As one of the PI3K p85 subunit family members, PIK3R2 suppresses the activation of the PI3K/AKT signaling pathway [13]. A recent study demonstrated that the gene coding for *PIK3R2* is targeted by miR-126 to inhibit the endothelial-to-mesenchymal transition of endothelial progenitor cells via regulation of the PI3K/AKT signaling pathway [13-14]. Moreover, miR-126 has been proposed as a therapeutic agent to treat undifferentiated thyroid carcinoma by targeting the *PIK3R2* gene and reducing p85β protein translation while lowering AKT kinase activity [15]. Cytokines in fibroblast-like synoviocytes lead to activation of the PI3K/AKT signaling pathway by binding to specific acceptors, thereby promoting migration and invasion of these cells. It has also been shown that the PI3K/AKT signaling pathway is involved in the pathogenesis of inflammation [16]. Therefore, modulation of the PI3K/AKT signaling pathway may yield therapeutic benefits for RA [17]. In our study, we aim to explore the effects of miR-126 on the proliferation and apoptosis of RASFs by targeting *PIK3R2* and regulating the PI3K-AKT signaling pathway.

## RESULTS

### Lentivirus packaging and infection efficiency observation

**Figure 1:**
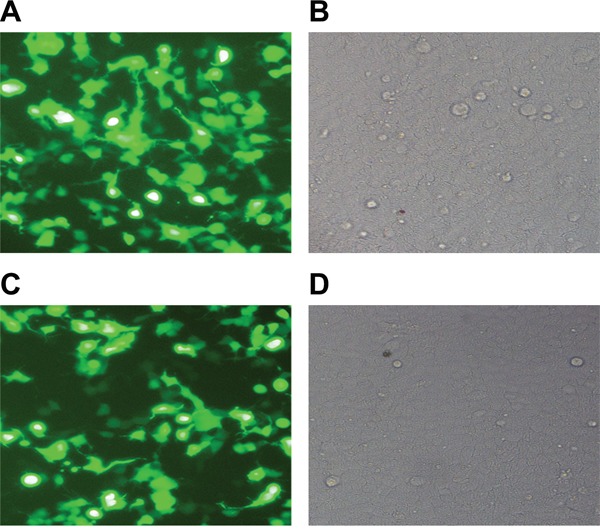
Observation of lentivirus packaging and infection efficiency Co-transfection of plasmids into the 293TN cells for 48 h under fluorescent **A.** and white light fields **B.** Infection of recombinant lentivirus into RASFs for 72 h under fluorescent **C.** and white light fields **D.** RASFs, rheumatoid arthritis synovial fibroblasts.

After co-transfection of plasmids into 293TN cells for 48 h, fluorescence microscopy was used to observe cell transfection efficiency, which reached ~90% (Figure 1A-1B). The multiplicity of infection (MOI) was set to 20, and the recombinant lentivirus was used to infect RASFs. After infection for 72 h, a fluorescent marker showed a higher gene transfection efficiency of ~90% (Figure 1C-1D).

### *PIK3R2* as a target gene of miR-126

**Figure 2:**
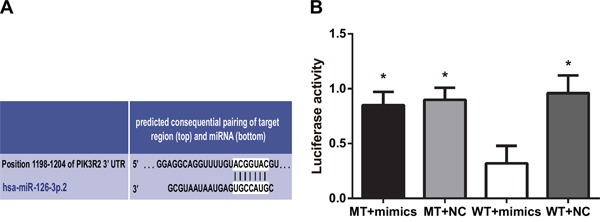
*PIK3R2* is a target gene of miR-126 **A.** TargetScan predicted that *PIK3R2* was a target of miR-126. **B.** Dual luciferase reporter gene activity detection. MiR-126, microRNA-126; MT, mutant; WT, wild type; NC, normal control; ^*^, compared with the WT + mimics group, *P* < 0.05.

Using the TargetScan Database (http://www.targetscan.org/), *PIK3R2* was found to be a potential miR-126 target gene (Figure 2A). RASFs were co-transfected with miR-126 mimics and wild-type (Wt-miR-126/PIK3R2) or mutant (Mut-miR-126/PIK3R2) recombinant plasmids. A double-luciferase reporter gene system showed that miR-126 mimics had no obvious effect on the luciferase activity intensity of mutant type Mut-miR-126/PIK3R2 plasmid, but significantly decreased that of the wild-type Wt-miR-126/PIK3R2 reporter plasmid (*P* < 0.05) (Figure 2B). The luciferase reporter assay indicated that miR-126 may bind to the 3′UTR of the *PIK3R2* gene, suggesting that *PIK3R2* is a miR-126 target.

### MiR-126 expressions in RASFs

**Figure 3:**
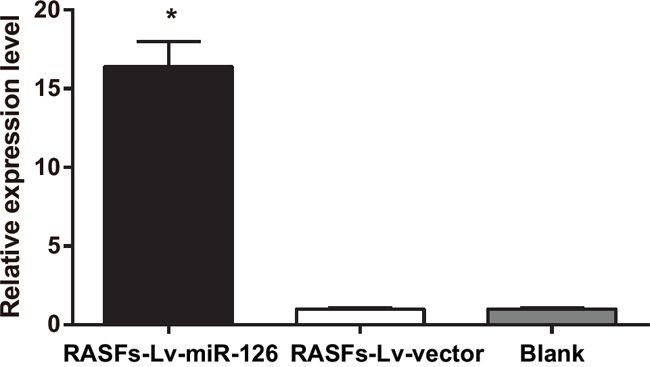
Real-time quantitative PCR detecting the expression level of miR-126 in RASFs PCR, polymerase chain reaction; Lv, lentivirus; miR-126, microRNA-126; RASFs, rheumatoid arthritis synovial fibroblasts; ^*^, compared with the blank group, *P* < 0.05.

After RASFs were transfected with lentivirus miR-126 expressing plasmid, the miR-126 expression in the RASFs-Lv-miR-126 group was significantly upregulated compared to that in the blank and empty vector control (RASFs-Lv-vector) groups (both *P* < 0.05). The difference in miR-126 expression was not statistically significant between the RASFs-Lv-vector and blank groups (*P* > 0.05) (Figure 3).

### MTT assay results

**Figure 4:**
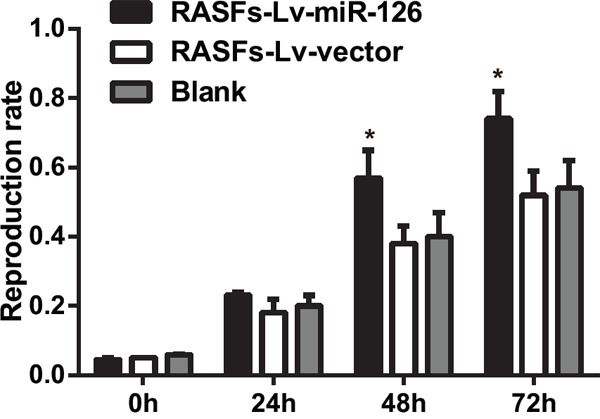
Overexpression of miR-126 increases proliferation of RASFs MTT assay detecting proliferation levels of RASFs after transfection in each group at different time. MTT, 3-(4,5-dimethylthiazol-2-yl)-2,5-diphenyltetrazolium bromide; RASFs, rheumatoid arthritis synovial fibroblasts; Lv, lentivirus; miR-126, microRNA-126; ^*^, compared with the blank group, *P* < 0.05.

Cell activity detection showed that an increased miR-126 expression promoted the growth and proliferative activity of RASFs. The proliferation rate of RASFs in the RASFs-Lv-miR-126 group at 24 h showed no difference compared with the blank group (*P* > 0.05). At 48 h and 72 h, the proliferation rates of RASFs were higher in RASFs-Lv-miR-126 group compared with that in the blank group (*P* < 0.05) (Figure 4). Therefore, overexpression of miR-126 can promote cell proliferation of RASFs.

### Flow cytometry analysis results

**Figure 5:**
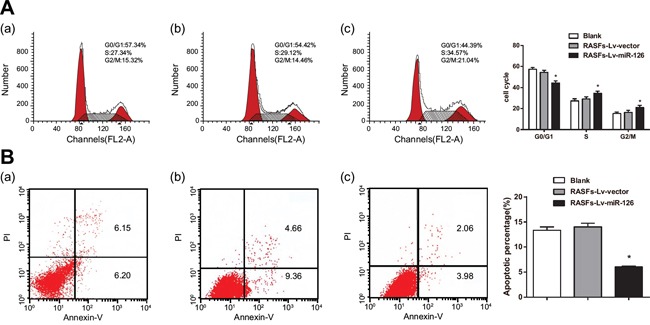
Overexpression of miR-126 inhibits RASF apoptosis **A.** Cell cycle and **B.** apoptosis of RASFs transfected with miR-126 and controls. RASFs, rheumatoid arthritis synovial fibroblasts; Lv, lentivirus; miR-126, microRNA-126; a, blank group; b, RASFs-Lv-vector group; c, RASFs-Lv-miR126 group; the left red area corresponds to the effective cell number in G1 phase, the intermediate blank area indicates the effective cell number in S phase, and the right red area shows the effective cell number in G2 phase; ^*^, compared with the blank group, *P* < 0.05.

Cell cycle analysis results showed that the proportions of cells in S phase and G2/M phase were increased in the RASFs-Lv-miR-126 group. The proportion of cells in each phase of the cell cycle in the RASFs-Lv-miR-126 group was different compared to cells in the RASFs-Lv-vector and blank groups (all *P* < 0.05) (Figure 5A). Results from cell apoptosis experiments showed fewer apoptotic cells in the RASFs-Lv-miR126 group than in the RASFs-Lv-vector and blank groups (both *P* < 0.05) (Figure 5B). These results suggest that miR-126 can inhibit RASF cell apoptosis by altering processes of the RASF cycle between the G0/G1 and S phase.

### Effects of miR-126 on PI3K-AKT pathway

**Figure 6:**
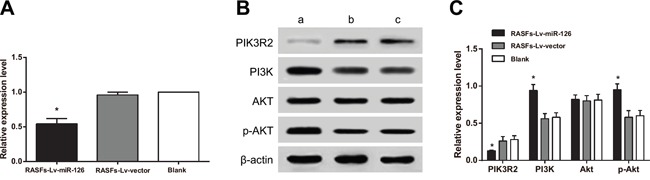
Overexpression of miR-126 inhibits *PIK3R2* and activates the PI3K/AKT signaling pathway **A.** Histogram showing the expression of *PIK3R2* mRNA detected by qRT-PCR. **B-C.** Western blot of PIK3R2, PI3K, AKT and p-AKT. miR-126, microRNA-126; qRT-PCR, quantitative real-time polymerase chain reaction; a, RASFs-Lv-miR126 group; b, RASFs-Lv-vector group; c, blank group; ^*^, compared with the blank group, *P* < 0.05.

We used qRT-PCR and western blot to test whether miR-126 in RASFs directly regulates *PIK3R2*. We found that the PIK3R2 mRNA (Figure 6A) and protein levels (Figure 6B) in the RASFs-Lv-miR126 group were decreased compared to those in the RASFs-Lv-vector and blank groups (both *P* < 0.05), indicating that miR-126 could target *PIK3R2* expression in RASFs. We further used western blot to test whether miR-126 could regulate the PI3K/AKT signaling pathway. The results showed that compared with the RASFs-Lv-vector and blank groups, total PI3K and p-AKT levels were increased in the RASFs of RASFs-Lv-miR126 group (all *P* < 0.05) (Figure 6C). On the other hand, total AKT levels showed no difference among the three groups. These results indicated that in RASFs, miR-126 expression inhibited *PIK3R2* expression to activate the PI3K-AKT pathway.

### Participation of *PIK3R2* in the miR-126-induced proliferation and apoptosis of RASFs

**Figure 7:**
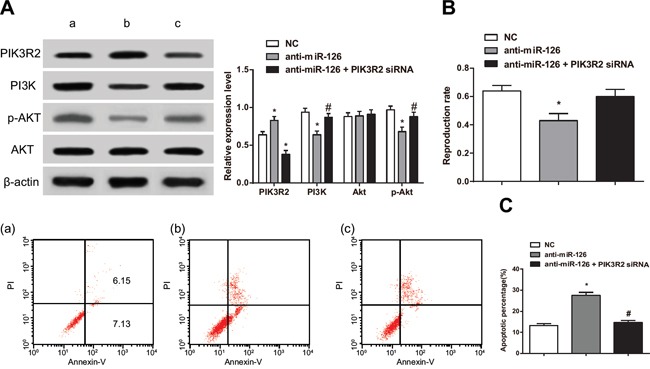
PIK3R2 participates in proliferation and apoptosis of RASFs induced by miR-126 **A.** Western blot detecting changes in expression for PIK3R2 and proteins in the PI3K/AKT signaling pathway. **B.** MTT assay measuring cell proliferation. **C.** Flow cytometry detecting apoptosis rate for cells in RASFs transfected with miR-126 and control cells. RASFs, rheumatoid arthritis synovial fibroblasts; miR-126, microRNA-126; MTT, 3-(4,5-dimethylthiazol-2-yl)-2,5-diphenyltetrazolium bromide; NC, normal control; a, blank group; b, anti-miR-126 group; c, anti-miR-126+ PIK3R2 siRNA group; ^*^, compared with the NC group, *P* < 0.05; ^#^, compared with the anti-miR-126 group, *P* < 0.05.

Using western blot, we found that the expression of PI3K and p-AKT after a single treatment of anti-miR-126 were decreased compared to the NC group (*P* < 0.05). On the other hand, PI3K and p-AKT expression after co-treatment with anti-miR-126 and PIK3R2 siRNA were increased compared to those in the single treatment with anti-miR-126 (all *P* < 0.05) (Figure 7A). MTT assay experiments showed that RASF proliferation was lower after a single treatment with anti-miR-126 compared to that in the NC group (*P* < 0.05) while increasing in the anti-miR-126 + PIK3R2 siRNA group (*P* < 0.05). However, no difference was detected when comparing the anti-miR-126 + PIK3R2 siRNA group with the NC group (*P* > 0.05) (Figure 7B). Flow cytometry analysis showed that the apoptosis rate of RASFs in the anti-miR-126 group was higher than that of the NC group (*P* < 0.05) On the other hand, the rate of apoptosis of the anti-miR-126 + PIK3R2 siRNA group was lower than that of the anti-miR-126 group (*P* < 0.05), but no different than that of the anti-miR-126 + PIK3R2 siRNA and NC groups (*P* > 0.05) (Figure 7C). These results indicated that *PIK3R2* directly participated in the proliferation and apoptosis induced by miR-126 in the RASFs.

## DISCUSSION

In our study, we aimed to explore the effects of miR-126 on the proliferation and apoptosis of RASFs. We hypothesized that by targeting *PIK3R2*, miR-126 regulates the PI3K-AKT signaling pathway in RASFs, thereby promoting proliferation and inhibiting apoptosis. Indeed, we found that increasing miR-126 expression levels can promote growth and proliferation of RASF cells while inhibit RASF cell apoptosis and functions on the process of RASF cycle from G0/G1 phase to S phase. MiRNAs act as important adjusters in several different biological processes, such as cell differentiation, metabolism, proliferation, and apoptosis [22]. MiRNAs are capable of regulating the expression of various target genes, which could eventually results in the change of biological function; and miR-126, located in intron 7 of the EGF-like domain 7 (EGFl7) gene [23], is a miR that is reported to be highly increased in endothelial cells and regulated by the transcription factors Ets-1 and Ets-2 [24, 25]. It is also associated with cell growth regulation in some organs, including gastric cancer, colorectal cancer, and liver carcinoma, through regulating different target genes, such as insulin receptor substrate, p85, PI3K, akt, and Crk [26-29]. In accordance with the results in this study, previous studies confirmed our results from the side: overexpression of miR-126 can increase the sensitivity of osteosarcoma cells to epigallocatechin-3-gallate by inducing apoptosis and functions as a tumor suppressor by inhibiting the expression of Sox2 [29, 30]. Moreover, miR-126 can promote neural stem cell proliferation and survival [31].

In our study, we conducted a luciferase reporter assay to test whether miR-126 binds to the 3′UTR of the *PIK3R2* gene. Our results showed that *PIK3R2* is a target of miR-126. We also showed that PIK3R2 mRNA and protein levels in RASFs transfected with Lv-miR126 were lowered compared to those in control cells. MiR-126-3p can target *PIK3R2* to suppress metastasis and angiogenesis in hepatocellular carcinoma [32]. MiR-126 also promoted the function of endothelial progenitor cells by suppressing *PIK3R2*. Furthermore, modulation of miR-126 has been proposed to yield therapeutic benefits for the treatment of deep vein thrombosis [33]. In addition, miR-126 might target *PIK3R2* to promote tumori genesis by regulating the PI3K/AKT signaling pathway [34].

We found increased PI3K and p-AKT levels in RASF cells transfected with Lv-miR126 compared to controls, suggesting that miR-126 expression inhibited *PIK3R2* expression, thereby activating the PI3K-AKT signaling pathway. The activated p-AKT protein is translocated into the cytoplasm or the nucleus where it phosphorylates a series of substrates that regulating protein synthesis and gene transcription [35]. As a serine/threonine protein kinase, AKT participates in the regulation of cell growth, proliferation and apoptosis. PI3K is the most prominent upstream activator of AKT, and increasing the activities of PI3K and AKT promotes tumor development [36]. The PI3K/AKT signaling pathway normally inhibits apoptosis in chondrocytes [37] and modulation of PI3K signaling has been proposed as a potential therapy in the treatment of immune-mediated arthritis [38]. Results from our previous studies indicated that the anti-survival and anti-invasion activities of trichostatin A in hypoxic RASFs were associated with PI3K/AKT signaling inactivation [39]. In our present study, we found that PIK3R2 is directly involved in the proliferation and apoptosis induced by miR-126 in RASFs.

Taken together, our results showed that miR-126 overexpression in RASFs reduced the expression of the *PIK3R2* gene and promoted RASF proliferation while inhibiting apoptosis. Although, Target Scan showed other miR-126 targets whose functional characterization will require further experiments, our results suggest that miR-126 inhibition might provide therapeutic benefits to patients with RA.

## MATERIALS AND METHODS

### Ethical approval

All the RA patients signed informed consent and the experiments were approved by the Ethical Committee of Zhujiang Hospital of Southern Medical University and complied with the guidelines and principles of the Declaration of Helsinki [18].

### Cell culture

Human joint synovial tissues were obtained from RA patients who underwent joint replacement surgery at the Zhujiang Hospital of Southern Medical University. Clinical diagnosis of RA patients met the American College of Rheumatology Criteria [19]. Synovial tissues were taken out intraoperatively and cut into pieces immediately under sterile conditions. The cut synovial tissues were subjected to digestion by 2.5 g/L trypsin at 37°C for 2 h and the digested synovial tissues were subjected to centrifugation to obtain RASFs. The RASFs were cultured at an incubator with 37°C and 5% CO_2_. When the cells were grown to a near confluence state, cells can be passaged and the 3^rd^-8^th^ generations of cells were used for subsequent experiments [20]. Lentivirus packaging cell line 293TN cells (System Biosciences, Mountain View, CA, USA) were adherently cultured for proliferation with dulbecco minimum essential medium (DMEM) containing 10% fetal bovine serum (FBS), and then formed the monolayer cells. The eugenic cells for 2 ~ 3 days were taken and cell suspension was diluted to 2×10^6^ ~ 2×10^7^/mL with serum-free medium; and 0.5 mL cell suspension was mixed with 0.4 mL calf serum and 0.1 mL dimethyl sulfoxide into 1 mL cell freezing tube, placed it at 4°C for 1 h and then at −20°C for 2 h, which was finally put into liquid nitrogen for cryopreservation.

### MiR-126 lentivirus vector construction, packaging and titer determination

According to miR-126 (human pre Has-miR-126) sequence information and pCDH-CMV-MCS-EFl-copGFP vector polyclone site information, the primers were designed as follows: upstream primer: 5′-TGTCTAGATGTGGCTGTTAGGCATGG(EcoRI)-3′ and downstream primer: 5′-ATAGGTACCA AGACTCAGGCCCAGGC(BamHI)-3′. EcoRI and BamHI and protection bases were added respectively in the restriction sites of upstream and downstream primers. The primers were synthesized by Shanghai Invitrogen biotechnology Co., Ltd. MiR-126 fragment was amplified by polymerase chain reaction (PCR) and the target gene fragment was recycled. The double-stranded DNA fragment and the linearized lentivirus vector plasmid (System Biosciences, Mountain View, CA, USA) cut by EcoRI and BamHI enzymes (TaKaRa, Japan) were connected by T4 DNA ligase (TaKaRa, Japan) under 16°C overnight. The connected product was transformed into DH5α competent cells (TaKaRa, Japan) and positive clones were picked and verified by sequencing. Lentivirus packaging cell line 293TN was subjected to recovery culture and Lipofectmaine 2000 (Invitrogen, USA) was used to co-transfect the mixture of expressing vectors and viral packaging into 293TN cells. After transfection for 24 h, the medium was changed by Dulbecco minimum essential medium (DMEM) medium containing 1% fatal bovine serum (FBS). The supernatants were collected after transfection for 72 h and were subjected to centrifugation at 5000 rpm/min for 10 min to remove cell precipitate. The centrifuged supernatants were filtered using 0.22 μm degerming filtration membrane and the filtered supernatants were sub-packaged and preserved, and virus titer determination was conducted using a gradient dilution method.

### Lentivirus infection

The experiment was divided into three groups: blank control group (blank group), empty vector control group (RASFs-Lv-vector) and RASFs-Lv-miR-126 group. Before infection, medium for RASF culture was changed with Polybrene containing complete medium and virus supernatant was added for culture. After infection for 6 h, the old medium was discarded and fresh complete medium was used to replace the old medium. After infection for 72 h, cells were placed under fluorescent microscope to observe infection efficiency. The cells were collected for cell transplantation and RNA extraction.

### Cell transfection

Cell grouping: anti-miRNA-126 group (single transfection of anti-miRNA-126), anti-miRNA-126 + PIK3R2 siRNA group (co-transfection of anti-miR-126 and PIK3R2 siRNA); normal control (NC) group (no treatment for RASF cell lines). One day prior to transfection, cells at a density of 2 × 10^5^ per well were seeded in 24-well plates. When the cells were grown to a density of 70%-80%, cells were confluent. Lipofectamine™ 2000 was used to respectively transfect miR-126 mimics, anti-miR-126 (miR-126 inhibitors) and PIK3R2 siRNA (Genepharma company, Shanghai, China) into RASFs. The specific method referred to the manual. The transfection of miR-126 mimics was used for luciferase activity measurement experiments; and the co-transfection of anti-miR-126 and PIK3R2 siRNA to RASFs were used to identify whether *PIK3R2* was directly involved in proliferation and apoptosis of miR-126-induced RASFs.

### Luciferase reporter vector construction and luciferase activity measurement

Both sides of the 3′ UTR (untranslated region) sequences of wild-type and mutant *PIK3R2* were added with Spe I and Hind III restriction sites, which was synthesized by Shanghai Sangon Biological Technology Co., Ltd. The sequences were cut by the double enzymes of SpeI and Hind III and the cut sequences were cloned into pMIR-REPORT™ Luciferase vector (Ambion, USA). After sequencing identification, the plasmids were named PIK3R2/WT and PIK3R2/mut. The plasmids were extracted in a strict accordance with TIANGEN biological TIANamp Genomic DNAKit instructions and the extracted plasmids were used for transfection using the Lipofectamine™ 2000 Transfection Reagent (Invitrogen company, US). The sample luciferase activity was detected with Dual-Luciferase Reporter Assay System (E1910) (Promega, US). After transfection for 48 h, the old medium was aspirated and discarded and the cells were washed with phosphate buffered saline (PBS) twice. A total of 100 μL of passive lysis buffer (PLB) was added into each well and the cells were slightly shaken at room temperature for 15 min to collect the cell lysates. The program pre-read was set at 2 s and the reading time at 10 s, and each injection of luciferase assay reagent II (LARII) and Stop&Glo^®^ reagent (Promega, USA) was 100 μL. The prepared LARII and Stop&Glo^®^ reagent and cell lysate added into luminescent tubes or plates (20 μL for each sample) into a bioluminescent detector to measure fluorescence.

### Real-time PCR detecting the expression levels of miR-126 and *PIK3R2*

The RNA of infected cells was extracted. The designed miR-126 reverse transcription primer miR-126-reverse transcription:

5′-GTCGTATCCAGTGCAGGGTCCGAGGTATTCGCACTGGATACGACCGCATT-3′ was subjected to reverse transcription to obtain the first strand of miR-126 cDNA. The first strand of U6 snRNA cDNA was obtained by reverse transcription of random primers containing 9 bases. After reverse transcription, the obtained cDNA was subjected to real-time quantitative PCR (qRT-PCR). The primer sequences of q-RT-PCR detection were shown in Table 1. The reaction conditions were as follows: 95°C pre-denaturation for 10 min, 40 cycles of 95°C denaturation for 10 s, 60°C annealing for 20 s, and 72°C extension for 34 s. ABI7300 fluorescence quantitative PCR instrument (ABI, USA) was used to analyze the Ct value in each group of amplification curves and the data were analyzed using 2^−ΔΔCt^ method [21]. 2^−ΔΔCt^ showed the target gene expression ratio relationship between the experimental and control groups, and the following formula was as follows: ΔΔCt = (Ct_target gene_-Ct_reference gene_)_experimental group_-(Ct_target gene_-Ct_reference gene_)_control group_. Ct was the number of amplification cycles when the real-time fluorescence intensity reached the set threshold and the amplification was in a logarithmic growth phase. The experiment was repeated for three times.

**Table 1:** The primer sequence of real-time polymerase chain reaction

	Sequence (5′-3′)
miR-126	F:CGGCTCGTACCGTGAGTAATAA
	R:GTGCAGGGTCCGAGGT
PIK3R2	F:GCACCACGAGGAACGCACTT
	R:CGTCCACTACCACGGAGCAG
U6	F:CGCTTCGGCAGCACATATAC
	R:CAGGGGCCATGCTAATCTT

Note: miR-126, microRNA-126; F, forward; R, reverse.

### 3-(4,5-dimethylthiazol-2-yl)-2,5-diphenyltetrazolium bromide (MTT) assay detecting cell proliferation

The RASFs, 48 h after viral infection, were taken and seeded in 96-well plates with 100 μL in each well containing 2 × 10^4^ cells. After being seeded, the RASFs were cultured under normal conditions. Four replicate wells were set in each group and cell activities in 24, 48 and 72 h were detected respectively: 20 μL of MTT solution (5 g/L) were added into each well and the cells were cultured for 4 h; the supernatant was discarded, 150 μL of dimethylsulfoxide (DMSO) were added, and the mixture was subjected to a low speed oscillation for 10 min using a shaker to fully dissolve the crystals; and a microplate reader was used to determine the absorbance at 570 nm. The above experiment was repeated for three times.

### Flow cytometry to detect cell cycle and apoptosis

After 48 h of transfection, the RASFs were collected and fixed in 75% ice ethylalcohol (precooled at −20°C) overnight at 4°C. After being centrifuged, these cells were separated from fixation fluid using ice PBS. Subsequently, with the addition of RNaseA, the cells were subjected to water bath in a dark condition for 30 min. Then propidiom iodide (PI) was added for coloring. After being mixed evenly, cell cycle stages were detected by red fluorescence recording during flow cytometry and the proportions of cells in G0-G1, S, and G2-M-phase were calculated. Each experiment was performed in triplicate.

Cell apoptosis assay was performed according to the operating manual, specifically including the following experimental procedures: Cells in each group were collected and washed with pre-cooled PBS for three times. After addition of 1 mL 1 × Annexin V binding buffer, the mixture was subjected to centrifugation and the supernatant was discarded. The remaining cells were re-suspend in 200 μL of binding buffer, and 10 μL of Annexin V-FITC (sigma, USA) and 5 μL of PI (5 mg/L) were added into the cell suspension, which was mixed gently and incubated in the dark for 30 min. Subsequently, flow cytometry (BD, USA) was used to analyze cell apoptosis. The results were represented as a scatter plot. Left lower quadrant (Q4): normal living cell (FITC-/PI-). Right lower quadrant (Q3): early cell apoptosis (FITC+/PI-). Right upper quadrant (Q2): advanced cell apoptosis and necrosis (FITC+/PI+). Apoptosis rate = Q3 + Q2.

### Western blot analysis

RASFs in each group were digested with protein lysate (Beyotime Biotechnology, Shanghai, China) to extract total protein. Bicinchoninic acid assay (BCA) kit (KeyGEN BioTECH, Nanjing, China) was used for protein quantitation. Firstly, the proteins were subjected to sodium dodecyl sulfate polyacrylamide gel electrophoresis (SDS-PAGE). Then, proteins were transferred to nitrocellulose membranes and sealed with 5% skim milk for 2 h. Diluted primary antibodies (PIK3R2: Santa Cruz Biotechnology, USA; PI3K: Santa Cruz Biotechnology, USA; AKT: Cell Signaling, USA; phospho-AKT: Cell Signaling, USA; β-actin: Cell Signaling Technology, USA) were added into the membranes, and the mixture was incubated at 4°C overnight. Then, the membranes were washed once with PBS twice with tris-buffered saline tween-20 (TBST). Horseradish peroxidase-labeled secondary antibodies were added and the mixture was incubated at room temperature for 2 h. After the membranes were washed, electrogenerated chemiluminescence (ECL) was used to develop the films, which were rinsed with pure water and left to dry. Scanning was used to record them.

### Statistical analysis

Data were analyzed using the statistical package for the social sciences (SPSS) version 21.0 (SPSS Inc.; Chicago, IL, USA). Continuous data were displayed as mean ± standard deviation, in which the differences between two groups were analyzed by the LSD-t test and among multiple groups by one-way analysis of variance (ANOVA) (homogeneity of variance was tested before analysis). A *P* < 0.05 was regarded as statistically significant.
